# Analgesic and Anti-Inflammatory Activities of Diethyl Ether and n-Hexane Extract of* Polyalthia suberosa* Leaves

**DOI:** 10.1155/2018/5617234

**Published:** 2018-01-23

**Authors:** Nelufar Yasmen, Md. Abdullah Aziz, Afsana Tajmim, Mst. Irin Akter, Amit Kumar Hazra, S. M. Mushiur Rahman

**Affiliations:** ^1^Department of Pharmacy, Jessore University of Science & Technology, Jessore 7408, Bangladesh; ^2^Department of Pharmacy, Stamford University Bangladesh, Dhaka 1217, Bangladesh

## Abstract

In folk medicine,* Polyalthia suberosa* is used as abortifacient, laxative, febrifuge analgesic, filler of tooth cavities, and anti-HIV drug and for rheumatism and various skin infections. The present study was directed to evaluate the analgesic and anti-inflammatory activities of diethyl ether and n-hexane extracts of* Polyalthia suberosa* leaves (PSDE and PSNH). A variety of tests including formalin-induced paw licking test, acetic acid induced writhing test, and tail immersion test were used to assess the analgesic activity. In addition, xylene-induced ear edema test was used to evaluate anti-inflammatory activity of PSDE and PSNH. PSDE and PSNH at 200 and 400 mg/kg doses expressed analgesic as well as anti-inflammatory activities in mice. In formalin-induced paw licking test, acetic acid induced writhing test, and xylene-induced ear edema test, the extracts exhibited significant inhibition (^⁎^*P* < 0.05 versus control) of pain and inflammation. Alternatively, in tail immersion test, PSDE 400 mg/kg showed significant (^⁎^*P* < 0.05 versus control) latency at 30 min but another tested sample had no significant latency. From this study, it could be shown that* Polyalthia suberosa* leaves may contain analgesic and anti-inflammatory agents which support its use in traditional medicine.

## 1. Introduction

Since the beginning of human being development, various medicinal plants are used as traditional medicines for remedial purpose. Plants possess the capability of producing biologically interesting and valuable chemical constituents [1, 2].

*Polyalthia suberosa* Roxb. is an evergreen shrub or small tree, which is naturally grown and widely distributed throughout Bangladesh, India, China, Malaysia, Myanmar, Sri Lanka, Philippine, Thailand, and Vietnam [3–5]. However, it is locally recognized as Jam debharu or Hamjam [6, 7]. It has corky barks, narrowly oblong or oblong-lanceolate, long acuminate, shining leaves, rusty tomentose, young branchlets, and so on. [8]. It is traditionally used as abortifacient, laxative, febrifuge analgesic, anti-HIV drug, and filler of tooth cavities [4, 6, 9]. Other documented folk medicinal uses of this plant are rheumatism and various skin infections [10]. In addition, seeds of this plant were considered as sedative, diuretic, and soporific [4]. A review of literature of this plant proposed that* Polyalthia suberosa* has a variety of activities including analgesic, cytotoxic, antibacterial, neuropharmacological, and antioxidant activity [3, 6, 10, 11].

Pain is an incapacitating accompaniment of many medical conditions [12] and according to the International Association for the Study of Pain (IASP), it is defined as “an unpleasant sensory and emotional experience associated with actual or potential tissue damage” [13, 14]. It always gives a warning signal and is primarily protective in nature but often causes a lot of discomfort and leads to many adverse effects [15]. Different biochemical mediators like prostaglandins, bradykinins, and substance P act on the nociceptors causing the sensation released by tissue injury and are considered as the immediate cause of pain. It can be either chronic or acute. Quick onset and short duration of pain that last for hours are regarded as acute pain, whereas chronic pain is characterized by persistent pain over a long period of time [16–18]. So, control of pain is one of the most important therapeutic priorities [12].

Inflammation is a permeative phenomenon that operates during severe impatience of homeostasis, such as infection, injury, and exposure to contaminants and this process is triggered by innate immune receptors and recognizes the pathogens to remove them [19]. This is a defense mechanism which is characterized by redness, pain, heat, swelling, and loss of function in the affected area [20, 21]. In the formation of inflammation, both the innate immune response and the acquired immune response are involved [22–25]. In mammalian cells, at least two isoforms COX-1 and COX-2 subsist, where COX-1 is distributed constitutively in almost all cell types, including platelets and those present in stomach, vascular endothelium, kidney, forebrain, and uterine epithelium regulated as a house keeping enzyme for various physiological functions. On the other hand, during tissue damage or inflammation in response to proinflammatory cytokines such as IL1, interferon gamma, and TNF-*α*, COX-2 is expressed and induced [26–29].

Both NSAIDs (Nonsteroidal Anti-Inflammatory Drugs) and steroidal drugs are used to treat pain and inflammation. However, long-term use of NSAIDs causes various adverse effects and damage liver, gastrointestinal tract, and so on. Besides, they initiate cardiovascular problems and renal failure [30–33]. Steroids may suppress the immune system and trigger erectile dysfunction, manic depression, hypertension, cramps and dizziness, appearance of dormant diabetes, skin atrophy, decreased bone density, stomach ulcers with possible perforation of the stomach wall, irregular menstruation, vision and allergic problems, and reduced wound healing [34, 35]. Moreover, opioid analgesics are used in pain management which have adverse effects for example, dependence, constipation, and respiratory problems [36].

For resisting these adverse effects, exploration of new analgesic and anti-inflammatory drugs are till now an arduous project and research is crucial to discover various alternatives. Plant based medicines may fulfill this requirement by providing nontoxic, more potent, efficacious, and safe drugs to treat pain and inflammation. Therefore, the aim of this study was to evaluate the analgesic as well as anti-inflammatory attributes of* Polyalthia suberosa* leaves.

## 2. Materials and Methods

### 2.1. Chemicals and Reagents

Diclofenac sodium was used as standard drug in this study and obtained from Square Pharmaceutical Ltd., Bangladesh; another standard drug, tramadol hydrochloride (TH), was purchased from Beximco Pharmaceutical Ltd., Bangladesh. All solvents used were of analytical grade and obtained from Merck, Germany.

### 2.2. Collection and Identification of Plant

At first with the help of a comprehensive literature review,* Polyalthia suberosa* (family Annonaceae) was selected for this investigation. Leaves of this plant were collected from Phultala, Khulna, Bangladesh, that is located at 22.9750°N, 89.4583°E. Species identification was verified by Naymur Rahman, Principal Scientific Officer at the Bangladesh National Herbarium, and the accession number is 44988. A dried specimen was deposited in the herbarium for future reference.

### 2.3. Extraction

After collection, the leaves were rinsed with water and dried in room temperature and then in air oven (LY-660, DONGGUAN LIYI Test Equipment Co. Ltd., China) at reduced temperature (not more than 40°C) to be suitable for grinding purpose. After that, leaves were ground into fine powder using high capacity grinding mill (Model 2000 LAB Eriez, USA) and then stored in air-tight container. Cold extraction procedure was used for preparing the extracts. Diethyl ether and n-hexane extracts were prepared by separately immersing 200 g powder of* Polyalthia suberosa* leaves in 2 L diethyl ether and n-hexane (Mark, Germany) for 7 days. Whatman number 1 filter papers were used to filter the liquid extracts. The filtrates were then dried in air to get solid residues. The extraction yields of PSDE and PSNH leaves were 8.34% (w/w) and 6.23% (w/w), respectively. However, both extracts were stored at 4°C for additional studies.

### 2.4. Experimental Animals

For the experiment, eighty* Swiss albino* mice of either sex, 6-7 weeks of age, weighing between 25 and 30 g, were purchased from International Center for Diarrhoeal Disease Research Bangladesh, Mohakhali, Dhaka, Bangladesh. Throughout the experiment, animals stayed under standard environmental conditions (temperature: 27.0 ± 1.0°C, relative humidity: 55–65%, and 12 h light/12 h dark cycle) with one-week adaptation before experiment. They were housed in cages made of polypropylene and had free access to feed and water ad libitum. All protocols for animal experiment were approved by the Institutional Animal Ethical Committee of Jessore University of Science and Technology, Jessore, Bangladesh.

### 2.5. Acute Toxicity Study

Acute toxicity results from a single exposure or multiple exposures of an ingredient within a short period (normally less than 24 h). This study was carried out according to the guidelines of Organization of Economic Cooperation and Development (OECD) for defining the half lethal dose (LD_50_) of the experimental samples. To conduct this study, fifteen mice were separated into three groups: control group and test groups (PSDE and PSNH), with each group having five animals. At various concentrations (100, 250, 500, 1000, 2000, 3000, and 4000 mg/kg body weight), the experimental samples were administered orally. At that moment, some parameters were observed such as mortality, diarrhea, noisy breathing, salivation, convulsion, injury, changes in locomotor activity, weakness, discharge from eyes and ears, coma, pain, aggressiveness, food or water rejection, or any other signs of toxicity in each group of animals for 5-6 h. And these parameters were monitored at the end of every hour also. Moreover, each group of animals was kept under observation for 2 weeks for the final assessment [37, 38].

### 2.6. Evaluation of Analgesic Activity

#### 2.6.1. Formalin-Induced Paw Licking Test

Formalin-induced paw licking test was performed according to Hunskaar and Hole [39]. Twenty mice were selected for this test and divided into four groups, having five mice in each group, and they were fasted for 16 h with water ad libitum. Control group, standard group, and test groups were treated with distilled water (10 mL/kg), diclofenac sodium (DS, 100 mg/kg), PSDE, and PSNH at 200 and 400 mg/kg, respectively. All of the treatment processes were done by oral gavage. Moreover, 1 h later of treatment, each mouse was injected with 20 *µ*L of 2.7% (v/v) formalin solution into the dorsal surface of left hind paw. Animals were observed for 5 min after injection, which was considered as acute phase. Again, they were monitored for 5 min after 20 min of injection which was defined as late phase. The percentage of inhibition of licking was calculated by the following formula.

(1) Inhibition %=1−Licking  time standard  or  extractsLicking  time normal  control×100

#### 2.6.2. Acetic Acid Induced Writhing Test

This test was performed according to Koster et al. [40]. Mice were kept unfed for 16 h with water ad libitum prior to the experiment and pretreated with extracts as mentioned before. DS (100 mg/kg) acted as standard or positive control, whereas distilled water acted as normal control. Each mouse was injected intraperitoneally with 0.7% (v/v) acetic acid at a dose of 10 mL/kg body weight after 45 min of respective treatment. The number of writhing responses was recorded for each animal during a 5 min period, which began after 15 min of acetic acid administration. To calculate the percentage of inhibition of writhing, the following formula was used.

(2) Inhibition %=1−No.  of  writhing standard  or  extractsNo.  of  writhing normal  control×100

#### 2.6.3. Tail Immersion Test

This test was conducted according to Toma et al. [41]. Central mechanism of pain or analgesic activity can be evaluated by this experiment. Thermal stimuli act as the generator of painful reaction through dipping the tail tip in hot water (55 ± 1°C). Mice were grouped and treated as described before. Here, tramadol hydrochloride (10 mg/kg) was used as reference drug. Basal reaction time was counted for each mouse after one hour of treatment. The counting was after 30, 60, 90, and 120 min of the respective treatment to determine the latency period. Moreover, each group was also monitored for latency period before 30 min of treatment. The animal which had more than 15 s latency periods was removed from the experiment and 15 s acts as cut-off point to avoid injury.

### 2.7. Evaluation of Anti-Inflammatory Activity

#### 2.7.1. Xylene-Induced Ear Edema

The method of Dai et al. [42] was used to evaluate xylene induced ear edema in mice. Twenty mice were divided into four groups: negative control group (distilled water, 10 mL/kg body weight), standard group (diclofenac sodium, DS, 100 mg/kg body weight), and test groups (200 and 400 mg/kg body weight), having five mice in each group. Negative control (10 mL/kg) received one dose of distilled water, where the standard group (100 mg/kg) treated with diclofenac sodium (DS) as well as test groups received PSDE and PSNH at 200 and 400 mg/kg orally. Each animal received 20 *μ*L of xylene on the anterior and posterior surfaces of the right ear lobe one hour after the particular treatment. The left ear was kept untreated and considered as control. After one hour of xylene application, the mice were sacrificed and 6 mm circular sections of the ears were taken by using a cork borer and weighed. The difference between weight of ear treated with xylene (right ear) and the weight of ear left untreated (left ear) was considered as the weight of xylene-induced edema. The percentage inhibition of ear edema was calculated by the following formula.

(3) Inhibition %=1−Weight  of  edema standard  or  extractsWeight  of  edema control×100

### 2.8. Statistical Analysis

All results are expressed as mean ± standard error of mean (SEM). All data were analyzed statistically by one-way ANOVA followed by Dunnett's *t*-test. Pairwise comparison of means among the groups was analyzed by one-way ANOVA followed by post hoc Tukey's HSD test.

In addition, the results of tail immersion test were analyzed by using repeated measure ANOVA (RM-ANOVA). *P* < 0.05 was considered to be statistically significant. SPSS software (version 17; IBM Corporation, New York, USA) was used to analyze all the data.

## 3. Results

From the acute toxicity study, sign of toxicity or mortality was not observed up to the high dose of 4000 mg/kg for PSDE as well as PSNH or control group. There was no change in food intake or other behaviors during 2-week observation period and was the same as prior to the experiment. This seemingly specified that the test groups did not express acute oral toxicity.

The effects of formalin-induced paw licking test of diethyl ether and n-hexane extracts of* Polyalthia suberosa *in mice are shown in Table 1. Tested samples (orally administered, 200 mg/kg and 400 mg/kg) suppressed the licking, persuaded by formalin in the biphasic pain response, those divided as the neurogenic pain response or the early phase, and the inflammatory pain response or the late phase. All the tested samples produced significant inhibition (^*∗*^*P* < 0.05 versus control) in late phase, whereas in acute phase only PSNH 400 mg/kg showed significant inhibition (30.31 ± 4.53%).

The result of acetic acid induced writhing test was summarized in Table 2. Diclofenac sodium (DS) 100 mg/kg was used as positive control and the inhibition of the acetic acid induced writhing was observed as 88.68 ± 3.61%. Orally administered tested samples expressed notable differences in the writhing occurrence and they antagonized the pain sensation. Tested samples showed significant (^*∗*^*P* < 0.05 versus control) inhibition of writhing and the highest percentage of inhibition was shown by PSNH 400 mg/kg (75.53 ± 8.36%).

The results of analgesic activity of* Polyalthia suberosa* diethyl ether and n-hexane extracts measured by tail immersion test were given in Table 3. At 30 min, PSDE 400 mg/kg showed significant (^*∗*^*P* < 0.05 versus control) latency but other tested samples had no significant latency. Tramadol hydrochloride (10 mg/kg) was found to be effective and significant (^*∗*^*P* < 0.05 versus control) at 30, 60, and 120 min.

The result of anti-inflammatory activity obtained from xylene-induced ear edema in mice is shown in Table 4. All of the groups showed significant (^*∗*^*P* < 0.05 versus control) inhibition of ear edema and differences of ear weight. Among the extracts, 65.92%  ± 1.62% is the highest value of inhibition that was provided by PSNH 400 mg/kg.

## 4. Discussion

Till now very limited toxicity studies have been carried out on plant derived products. However, not only adverse effects of any plant material but also its safe doses can be determined by acute oral toxicity test [37].

In this study, LD_50_ of the plant extracts could not be obtained, as no mortality was observed up to the dose as high as 4000 mg/kg and the extracts were found to be safe with a broad therapeutic range. Therefore, two comparatively high doses (200 and 400 mg/kg) for both PSDE and PSNH were used for* in vivo* doses.

The formalin-induced paw licking test is a trustworthy method for investigating nociception as well as possible mechanism of analgesia. Two distinct mechanisms are associated with the biphasic behavior of pain in this test [39]. The early phase is considered as neurogenic pain phase which is induced by direct stimulation through formalin that starts instantly after the formalin solution has been injected and lasts for 5–10 min. The late phase shows inflammatory pain which arises from the spinal neuro-hyperactivity involving various mediators which are started by the C-fibers. Peripherally acting drugs act only on the late phase but centrally acting drugs show good response in both phases [16, 43, 44]. The results of the formalin test stated that PSDE and PSNH work on late phase and inhibit inflammatory mediators, whereas in acute phase PSNH 400 mg/kg showed significant inhibition. Maximum inhibition was exhibited by PSDE 400 mg/kg (62.75 ± 11.97%).

Globally acetic acid induced writhing test is being primarily used for the assessment of antinociceptive activity of natural composites [45, 46]. The release of different endogenous noxious mediators such as bradykinin, serotonin, histamine, and substance P is induced by acetic acid. Contraction of abdominal muscle goes together with expansion of the forelimbs and body elongation that characterize the pain which is brought about by acetic acid [2, 46, 47]. Local peritoneal receptors as well as prostaglandin pathways are thought to be responsible for abdominal contraction [48–51]. In this study, PSDE and PSNH showed significant inhibition of acetic acid induced writhing and this may occur due to the inhibition of endogenous mediators or blockage of prostaglandin pathways.

The tail immersion test is an important acute pain model [52] and it assesses the centrally acting analgesic and opioid receptor agonist. Opioids analgesic activities are ascertained through spinal (*δ*2, *σ*2, and *κ*1) and supraspinal (*δ*1, *σ*1, and *κ*3) receptors [53]. The opioid *µ* receptor demonstrates more sensitivity to thermal induced nociceptive test like tail immersion test. Opioids can start their antinociceptive activity in both acute and late phases of pain model [37, 54]. Table 3 shows that plant extracts showed less effect on inhibition of tail withdrawal and there was no significant latency period, except PSDE 400 mg/kg at 30 min (*P* < 0.05). So, there is less possibility of the involvement of opioids. Now it is necessity for broad studies to reveal the exact pain inhibitory mechanism of actions of the plant extracts.

Inflammation has four cellular processes, which are changes in blood flow by changing in smooth muscle cell function that are accountable for vasodilatation, alteration of the vascular permeability, migration phagocytic leukocytes to the site of inflammation, and phagocytosis [55]. Xylene-induced ear edema test is done as acute inflammatory test. In addition, xylene can release inflammatory mediators such as bradykinin, histamine, and serotonin. These mediators are responsible for edema as they enhance vascular permeability and improve vasodilation [56]. Fluid accumulation occurs at the treatment site, which is shown by the xylene-induced ear edema test and inhibition of this fluid accumulation is considered as anti-inflammatory effect [57]. Topical steroids or nonsteroidal anti-inflammatory agents inhibit phospholipase A2 which can be evaluated by the xylene-induced ear edema test [37]. Here, PSDE and PSNH produced significant inhibition of ear edema that may be due to the blockage of phospholipase A2, reduction of vascular permeability, and vasodilation. But extensive study is required to assure the exact mechanism, through which the extracts suppressed edema.

## 5. Conclusion

From the present study, it could be proposed that the diethyl ether and n-hexane extracts of* Polyalthia suberosa* leaves might possess analgesic and anti-inflammatory activities. Now it is under investigation of isolating and determining the active constituents and structures of* Polyalthia suberosa* leaves. However, further quantifiable studies are now essential to categorize the particular mechanism which is responsible for the analgesic and anti-inflammatory activities of* Polyalthia suberosa* leaves. At last but not least, to be a safe therapeutic agent, not only acute oral toxicological evaluation but also genotoxicity study of this plant should be conducted.

## Figures and Tables

**Table 1:** Effect of PSDE and PSNH in formalin-induced paw licking test.

Group	Dose	Acute phase		Late phase	
		Licking time (s)	Inhibition (%)	Licking time (s)	Inhibition (%)
Control	10 mL/kg	84.40 ± 4.64	0.00 ± 0.00	59.00 ± 3.91	0.00 ± 0.00
DS	100 mg/kg	58.00 ± 7.18^*∗*^	31.49 ± 9.71^*∗*^	12.20 ± 5.28^*∗*^	81.14 ± 7.44^*∗*^
PSDE	200 mg/kg	71.00 ± 6.05	16.89 ± 7.51	31.00 ± 7.45^*∗*^	47.70 ± 10.60^*∗*^
PSDE	400 mg/kg	63.20 ± 3.20	26.10 ± 3.67	23.60 ± 8.39^*∗*^	62.75 ± 11.97^*∗*^
PSNH	200 mg/kg	64.20 ± 9.65	25.04 ± 11.01	32.40 ± 8.23^*∗*^	44.95 ± 12.95^*∗*^
PSNH	400 mg/kg	59.80 ± 4.44^*∗*^	30.31 ± 4.53^*∗*^	25.40 ± 6.45^*∗*^	52.59 ± 9.24^*∗*^

All of the experimental values are denoted as mean ± SEM. ^*∗*^*P* < 0.05 versus control (Dunnett's *t*-test).

**Table 2:** Effect of PSDE and PSNH in acetic acid induced writhing test.

Group	Dose	Writhing number	Inhibition (%)
Control	10 mL/kg	22.00 ± 2.62	0.00 ± 0.00
DS	100 mg/kg	2.60 ± 0.74^*∗*$^	88.68 ± 3.61^*∗*$^
PSDE	200 mg/kg	12.80 ± 3.30^*∗*^	44.08 ± 15.78^*∗*^
PSDE	400 mg/kg	10.20 ± 3.29^*∗*^	57.86 ± 12.37^*∗*^
PSNH	200 mg/kg	9.20 ± 1.01^*∗*^	59.86 ± 6.23^*∗*^
PSNH	400 mg/kg	5.80 ± 2.03^*∗*^	75.53 ± 8.36^*∗*^

All of the experimental values are denoted as mean ± SEM. ^*∗*^*P* < 0.05 versus control (Dunnett's *t*-test). ^$^*P* < 0.05 versus PSDE 200 mg/kg (pairwise comparison by post hoc Tukey's HSD test).

**Table 3:** Analgesic effect of PSDE and PSNH in tail immersion test.

Group	Dose	Latency period (s)				
		0 min	+30 min	+60 min	+90 min	+120 min
Control	10 mL/kg	1.4680 ± 0.14	1.42 ± 0.29	1.41 ± 0.16	1.74 ± 0.16	1.20 ± 0.30
TH	10 mg/kg	1.7020 ± 0.14	6.96 ± 1.50^*∗*^	6.68 ± 2.00^*∗*^	4.48 ± 2.19	4.07 ± 1.21^*∗*^
PSDE	200 mg/kg	1.5180 ± 0.14	2.87 ± 0.33	3.74 ± 1.31	2.61 ± 0.62	2.06 ± 0.27
PSDE	400 mg/kg	1.6540 ± 0.21	4.47 ± 0.67^*∗*^	3.75 ± 1.89	2.17 ± 0.25	2.64 ± 0.38
PSNH	200 mg/kg	1.6540 ± 0.25	2.71 ± 0.52	2.01 ± 0.50	1.83 ± 0.14	1.77 ± 0.28
PSNH	400 mg/kg	1.2920 ± 0.10	3.12 ± 0.63	2.56 ± 0.30	2.41 ± 0.13	2.07 ± 0.11

Latency period evaluated from this study is represented as mean ± SEM. 0 min indicates 30 min before treatment and +30, +60, +90, and +120 mean after 30 min, 60 min, 90 min, and 120 min of treatment, respectively. Tests of within-subjects effects reveal that for the factor “time” calculated *F* = 9.30 for all methods and *P* value = 0.000 in every case. So, time is highly significant at any level of significance. ^*∗*^*P* < 0.05, versus control. Repeated measure analysis of variance with Dunnett's multiple comparison was performed to analyze this data set.

**Table 4:** Effect of PSDE and PSNH in xylene-induced ear edema in mice.

Group	Dose	Ear weight difference (mg)	Inhibition (%)
Control	10 mL/kg	32.60 ± 2.65	0.00 ± 0.00
DS	100 mg/kg	10.00 ± 0.54^*∗*$^	68.33 ± 3.61^*∗*$^
PSDE	200 mg/kg	18.20 ± 1.24^*∗*^	42.70 ± 5.57^*∗*^
PSDE	400 mg/kg	12.20 ± 2.26^*∗*^	62.67 ± 4.65^*∗*^
PSNH	200 mg/kg	15.80 ± 2.69^*∗*^	48.54 ± 12.00^*∗*^
PSNH	400 mg/kg	11.00 ± 0.70^*∗*$^	65.92 ± 1.62^*∗*$^

Ear weight differences are denoted as mean ± SEM. ^*∗*^*P* < 0.05 versus control(Dunnett's *t*-test). ^$^*P* < 0.05 versus PSDE 200 mg/kg (pairwise comparison by post hoc Tukey's HSD test).
